# Молекулярные и клеточные механизмы старения: современные представления (обзор литературы)

**DOI:** 10.14341/probl13278

**Published:** 2023-11-11

**Authors:** Р. К. Михеев, Е. Н. Андреева, О. Р. Григорян, Е. В. Шереметьева, Ю. С. Абсатарова, А. С. Одарченко, О. Н. Оплетаева

**Affiliations:** Национальный медицинский исследовательский центр эндокринологии; Национальный медицинский исследовательский центр эндокринологии; Московский государственный медико-стоматологический университет им. А.И. Евдокимова; Национальный медицинский исследовательский центр эндокринологии; Национальный медицинский исследовательский центр эндокринологии; Национальный медицинский исследовательский центр эндокринологии; Национальный медицинский исследовательский центр эндокринологии; Кубанский государственный технологический университет

**Keywords:** старение, омиксный, антивозрастной, теломераза, геростимуляторы, геропротекторы

## Abstract

Старение — запрограммированный на генетическом и эпигенетическом уровнях патофизиологический процесс, скорость которого определяется соотношением между факторами повреждения, с одной стороны, и факторами репарации организма — с другой. Старение является крайне актуальной проблемой не только в научном, но и в социально-демографическом плане. Согласно последним отчетам Всемирной организации здравоохранения (WHO, ВОЗ), общее число людей старше 60 лет продолжает неуклонно расти. Их общее число к 2020 г. уже достигло отметки в 1 млрд человек; при сохранении существующей тенденции к 2030 г. число пациентов достигнет отметки в 1,4 млрд, к 2050 г. — в 2,1 млрд человек. Отсутствие на сегодняшний день универсальной теории старения является поводом для научно-клинических изысканий с привлечением фундаментальных и клинических специалистов, в т.ч. эндокринологов. Ключевой интерес представляют последние данные о потенциальных источниках «anti-age лекарств»: натуральных и синтетических регуляторах теломераз, мезенхимальных стволовых клетках и т.д. Целью данного обзора литературы является освещение современных омиксных (геномных, протеомных, метаболомных) теорий старения, а также потенциальных способов таргетной (прицельной) профилактики и терапии возраст-ассоциированных заболеваний в рамках концепции персонализированной медицины. Данный обзор носит описательный характер, не преследует цель систематического обзора и метаанализа, а также рекламные цели. Представлена база данных PubMed за период с 1979 по 2022 гг.

## Abstract (en)

Ageing (as known as eldering, senescence) is a genetically and epigenetically programmed pathophysiological process. Velocity of biological ageing is defined as balance between alteration and reparation of body structures. According to last World Health Organization (WHO) highlights ageing still stays an extremely actual scientific, social and demographic problem: in 2020 total number of people older than 60 years and older was 1 billion people; in 2030 future number may be 1,4 billion people, in 2050 — 2,1 billion people. Absence of single universal theory of aging nowadays is reason for scientifical and clinical collaboration between biologists and doctors, including endocrinologists. Designing of potentially effective newest anti-ageing strategies (such as natural/synthetic telomerase regulators, mesenchymal stem cells etc.) is of interest to scientific community. The aim of present article is a review of modern omics (genomic, proteomic, metabolomic) ageing mechanisms, potential ways of targeted prevention and treatment of age-related disease according to conception of personalized medicine. Present review is narrative, it does not lead to systematic review, meta-analysis and does not aim to commercial advertisement. Review has been provided via PubMed article that have been published since 1979 until 2022.

## ГЛОССАРИЙ

Caenorhabditis elegans — почвенный круглый червь (нематода) размером ~1 мм, модельный организм для лабораторных биологических исследований.

Аноикис — разновидность апоптоза, наступающего при нарушении прикрепления клеток к другим клеткам или какой-либо поверхности.

Ацетилирование — органическая реакция присоединения остатка уксусной кислоты CH₃CO.

Геномный — имеющий отношение к изменениям совокупного наследственного материала организма (генома).

Героиндикатор — метаболит, позволяющий определить степень старения или эффективности мероприятий по противодействию процессу старения.

Гликирование — биохимическая реакция между восстанавливающими углеводами (глюкоза, фруктоза) и свободными аминогруппами белков, липидов, нуклеиновых кислот.

Дезамидирование — органическая реакция отщепления амидной (NH2-) функциональной группы от какого-либо соединения.

Деметилирование — органическая реакция отщепления метильной (CH3-) функциональной группы от какого-либо соединения.

Метаболомный — имеющий отношение к совокупности конечных продуктов обмена веществ в клетке, ткани, органе или организме (метаболому).

Метилирование — органическая реакция присоединения метильной группы CH₃-.

Нейросупрессор — вещество, подавляющее нейрональную активность.

Нокаутный — связанный с целенаправленным «выключением» или заменой какого-либо гена в ходе генно-инженерных манипуляций.

Омиксный — основанный на совокупности наследственного материала, продуцируемых белков и метаболитов технологий.

Протеомный — имеющий отношение к совокупности белков, производимых клеткой, тканью, органом или организмом.

Рибозилирование — биохимическая реакция присоединения пятиуглеродных моносахаридов к др. молекуле.

Сумоилирование (от англ. “Small Ubiquitin-like Modifier” — малые убиквитин-подобные белки-модификаторы) — регуляторная биохимическая реакция присоединения белков с низкой молекулярной массой (10–15 кДа) к остатку лизина.

Убиквитинирование (от англ. ubiquitous — «вездесущий») — биохимическая реакция присоединения низкомолекулярных белков (8 кДа) к другим белкам с целью контроля их взаимодействия и осуществления их деградации.

Экстрагирование — процесс извлечения какого-либо вещества с применением растворителя (экстрагента).

## ВВЕДЕНИЕ

С точки зрения термодинамики старение является процессом достижения биологическим объектом своего стационарного, т.е. мертвого, состояния, характеризующегося максимальной энтропией [1–3]. В соответствии с этим под старением мы понимаем универсальный механизм накопления изменений на различных уровнях живого, приводящий к постепенному снижению жизненной активности и, как следствие, смерти [4].

Старение — это неизбежный по своей сути, непрерывный биологический процесс, который заложен в нашем геноме. Скорость старения определяется соотношением химических, физиологических и патофизиологических процессов повреждения, с одной стороны, и процессов репарации — с другой. Старение является крайне актуальной проблемой не только в научном, но и в социально-демографическом плане. По данным Всемирной организации здравоохранения (ВОЗ), общее число людей старше 60 лет неуклонно растет. Их общее число к 2020 г. уже достигло отметки в 1 млрд человек; при сохранении существующей тенденции к 2030 г. число пациентов достигнет отметки в 1,4 млрд, к 2050 г. — в 2,1 млрд человек [5].

Это обуславливает актуальность исследования проблемы старения. Поиск источника вечной молодости начинается с незапамятных времен, о чем свидетельствует историко-культурное наследие, доставшееся нам от «отцов античной медицины», средневековых алхимиков, экспериментаторов Нового Времени [6]. Одним из самых харизматичных памятников фундаментальной геронтологии стало произведение венецианского аристократа Луиджи Корнаро (1467–1566), описавшего свой опыт по применению принципов здорового образа жизни, благодаря применению которых Корнаро достиг возраста 99 лет, в то время как средняя продолжительность жизни в то время составляла около 30 лет [7].

В настоящее время достигнуты существенные успехи в изучении биологии старения, в частности, методах расчета биологического возраста вне зависимости от хронологического [8]. В современной науке выделяют несколько теорий старения, схематически их классификация приведена на рис. 1.

**Figure:**
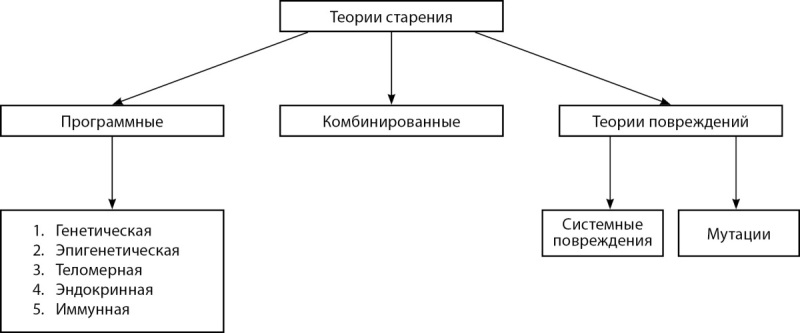
Рисунок 1. Классификация современных теорий старения.

Целью данной публикации является попытка провести описательный обзор современных представлений о молекулярных и клеточных механизмах старения, а также неординарных подходов к anti-age терапии.

## ПОНЯТИЕ О ПРОГРАММНЫХ ТЕОРИЯХ СТАРЕНИЯ

В центре внимания программных теорий находятся механизмы, последовательная и прогрессирующая активация которых неизбежна в организмах [9]. Среди программных теорий выделяют генетическую, эпигенетическую, теломерную, эндокринную и иммунную. Краткая информация об этих теориях изложена в следующих разделах данной статьи.

## ГЕНЕТИЧЕСКАЯ И ЭПИГЕНЕТИЧЕСКАЯ ТЕОРИИ СТАРЕНИЯ

Рассмотрение программных версий старения логично начинать с генетической и эпигенетической теорий. Согласно генетической теории, причиной старения являются точечные мутации в генах, определяющих продолжительность жизни. Эта гипотеза была сформулирована относительно давно: так, одна из первых работ в этой области вышла в свет в 1990 г. в журнале Science. В ней говорилось о том, что мутация в гене age-I у нематод Caenorhabditis elegans приводит к увеличению продолжительности жизни особей данного вида в среднем на 65% (25,3 vs. 15,0 дня), а максимальное увеличение продолжительности жизни составляет 110% (46,2 vs. 22,0 дня). Стоит отметить, что данная мутация увеличивала продолжительности жизни, не затрагивая длительность периода эмбриогенеза и поведение червя [9]. Полученные результаты оказались сенсационными для своего времени; высказывались даже гипотезы о нахождении «гена бессмертия». При подробном изучении было установлено, что продукты экспрессии age-1 являются не универсальными геропротекторами (веществами, защищающими от старения), а лишь температурозависимыми компонентами метаболического инсулиноподобного сигнального пути [10][11].

На сегодняшний день достоверно доказан вклад нарушения экспрессии большого числа генов в развитие возраст-ассоциированных заболеваний (табл. 1).

**Table:** Таблица 1. Гены, потенциально влияющие на скорость старения организма (Bin-Jumah et al., 2022) [12] Примечание. Курсивом обозначены названия генов, обычным шрифтом — метаболиты, оказывающие активирующее (активаторы) и ингибирующее (ингибиторы) действие на теломеразу.

Ген	Экспрессируемый белок	Физиологическая роль	Клинические проявления нарушения экспрессии
APOE	Аполипопротеин Е	Регуляция липидного обмена, агрегация тромбоцитов, пролиферация лимфоцитов	Атеросклероз, ишемическая болезнь сердца, болезнь Альцгеймера
P53	Фактор-супрессор злокачественных опухолей	Фактор транскрипции	Рост злокачественных образований
SIRT1	Сиртуин 1	Поддержание энергетического обмена, репарация ДНК	Старение, развитие злокачественных новообразований, дислипидемия
DAF-16	Фактор транскрипции FOXO1	Фактор транскрипции	Нарушение митоза, апоптоза клеток
CHRNA3	Нейрональная субъединица рецептора ацетилхолина альфа-3	Инактивация психоактивных веществ (ПАВ): никотина и этанола; поддержание активности ЦНС	Заболевания периферических артерий, хроническая обструктивная болезнь легких
SH2B3	Адаптерный белок 3	Адаптация к неблагоприятным факторам	Рост злокачественных новообразований, инсулинорезистентность
CDKN2A	Циклин-зависимый ингибитор киназы 2	Контроль митотического цикла	Сахарный диабет 2 типа, ожирение, дистрофия кардиомиоцитов
ELOVL2	Удлинение длинноцепочечных жирных кислот	Синтез арахидоновой кислоты, участие в воспалительных реакциях	Глюкозотоксичность, липотоксичность
WRN	Белок Вернера	Репарация ДНК, поддержание стабильности ДНК	Преждевременное старение, катаракта, атеросклероз, остеопороз, злокачественный рост
PON1	Пароксоназа 1	Инактивация гомоцистеина, защита от атеросклероза	Сердечно-сосудистые заболевания
SOD2	Супероксиддисмутаза 2	Антиоксидантное действие	Болезнь Альцгеймера
LMNA	Белок ламин А	Антиоксидантное действие	Ускоренное старение
CETP	Белок-переносчик эфиров холестерина	Образование липопротеинов	Дислипидемия, рост злокачественных новообразований
APOC3	Аполипопротеин С3	Транспорт липидов, углеводный обмен, передача нейронального импульса	Инсулинорезистентность, кальцификация коронарных артерий и восходящего отдела аорты
MTP	Микросомальный белок-переносчик триглицеридов	Синтез холестерина, транспорт липидов,	Метаболический синдром
PIK3CA	Фосфатидилинозитол-3-киназа	Дифференцировка нейронального импульса	Рост злокачественных новообразований молочной железы
DAF-2	Рецептор к ИФР-1	Анаболический и митогенный эффект	Болезнь Паркинсона, инсулинорезистентность, сахарный диабет 2 типа
PIMT	L-изоаспартилметилтрансфераза	Внутриклеточная передача сигнала	Болезнь Паркинсона, болезнь Альцгеймера
GH1	Гормон роста	Стимуляция роста и развития живых организмов, посредством ИФР-1	Старение, хроническая болезнь почек
KLOTHO	Белок альфа-клото	Усиление экскреции фосфора, кардио- и нефропротекция	Хроническая болезнь почек, сердечно-сосудистые заболевания, снижение продолжительности жизни

Однако наличие того или иного аллеля или точечной мутации гена далеко не всегда тождественно каким-либо фенотипическим проявлениям, что выходит за рамки менделевского наследования. В отсутствие мутаций число вариантов экспрессии тех или иных генов также крайне велико. Во многом это обусловлено так называемыми эпигенетическими факторами — «надстройками», регулирующими синтез белка с тех или иных участков ДНК [13]. Последние могут подвергаться ацетилированию [14] и метилированию [15], сумоилированию [16], убиквитинированию [17] и рибозилированию [18]; все эти процессы могут влиять на синтез конкретных белков. Указанные процессы также обладают героиндикаторными и геропротекторными эффектами [19], в связи с чем на их основании были разработаны многочисленные концепции «эпигенетических часов» — тончайших механизмов генного аппарата, позволяющих оценить биологический и хронологический возраст организма.

Одними из самых общеизвестных эпигенетических возрастных механизмов являются так называемые механизмы Хорвата (Steve Horvath) и Ханнума (Gregory Hannum), в основе которых лежит метилирование ДНК — биологический процесс, непосредственно ассоциированный со старением. Согласно концепции, предложенной Хорватом, возраст организмов положительно коррелирует с числом метилированных CpG-динуклеотидов в ДНК с поправкой на погрешность в ~3,6 года [20]. С целью увеличения доступности данной терапии был разработан онлайн-калькулятор, который позволяет с использованием искусственного интеллекта на основании степени метилирования ДНК ориентировочно оценить биологический возраст и прогнозировать смертность от различных причин [21]. В свою очередь, так называемые «часы Ханнума» аналогично позиционируются как идентичные в плане эффективности и точности прогнозирования методике Хорвата [22]. По мнению авторов, эпигенетические калькуляторы на основе часов Ханнума и Хорвата станут в будущем ключевыми инструментами для формирования прогностических моделей функционирования клеток, тканей, органов и организмов в целом.

## ТЕЛОМЕРНАЯ ТЕОРИЯ

В 1961 г. профессор Леонард Хейфлик (род. 1928) установил предел деления человеческих клеток — он соответствовал примерно 50 делениям в культуре in vitro. 10 лет спустя для подтверждения идей Хейфлика советским ученым Алексеем Матвеевичем Оловниковым (1936–2022) была выдвинута теория маргинотомии, объяснявшая старение недорепликацией последовательностей ДНК на концах хромосом (теломерных участков) в ходе деления. В 1998 г. вывод А.М. Оловникова был экспериментально подтвержден американскими исследователями (в т.ч. Хейфликом), которым удалось преодолеть предел Хейфлика путем активации фермента теломеразы [23]. Мировое признание данного открытия всемирным ученым сообществом и Шведской королевской академией наук увенчалось награждением группы ученых в составе Элизабет Блекберн, Кэрол Грейдер и Джека Шостака Нобелевской премией по физиологии и медицине 2009 г. [24].

Теломеры — это концевые структуры на концах хромосом, представляющие собой совокупность тандемных повторов из 6 нуклеотидов (TTAGGG) и в совокупности с защитными белками-шелтеринами (от англ. shelter — убежище) обеспечивающие стабильность генетической информации. Теломеры являются важнейшим индикатором жизненного цикла клетки, так как при каждом делении происходит их укорочение, при критическом укорочении теломер наступает программная гибель клетки (апоптоз) [25]. Особняком стоят клетки с высокой активностью фермента теломеразы. Данный фермент, относящийся к семейству ДНК-полимераз, обладает уникальной способностью замедлять укорочение теломер, обуславливая феномен «клеточного бессмертия» (в частности, у стволовых и злокачественных клеток) [26].

Теоретически фармакологические агенты — регуляторы теломераз могут быть использованы для профилактики и лечения возраст-ассоциированных заболеваний [27]. Среди таких регуляторов выделяют синтетические активаторы и ингибиторы теломераз, натуральные активаторы и ингибиторы теломераз.

Активаторы теломераз теоретически могут применяться у пациентов с длительно не заживающими раневыми дефектами, повреждениями опорно-двигательного аппарата, при профилактике нейродегенеративных заболеваний. Ингибиторы теломераз могут стать основой для создания противоопухолевых препаратов новейшего поколения [27]. Кратко сведения об известных регуляторах теломераз приведены в таблице 2 [27].

**Table:** Таблица 2. Потенциальные агенты-регуляторы (активаторы/ингибиторы) теломераз (по Fragkiadaki P et al., 2022) [27] Примечание. Курсивом обозначены названия генов, обычным шрифтом — названия фармакологических агентов, оказывающие активирующее (активаторы) и ингибирующее (ингибиторы) влияние на активность теломеразы.

Наименование потенциальной группы регуляторов теломеразы и ее происхождение	Наименование потенциального агента-регулятора теломеразы	Механизм действия
Синтетические ингибиторы теломеразы	BIBR1532 (нафталеновое производное бензойной кислоты)	Торможение образования тандемных повторов ТТАГГГ
Производные дигидропиразола	Блокада субъединицы теломеразы hTERT
Сибилинин	Подавление активности каталитической субъединицы теломеразы; подавление экспрессии гена hTERT в злокачественных клетках молочной железы
Производные куркуминоидов	Снижают аффинность теломеразы к учаcткам теломер
Производные этилсульфонилфторида	Потенциально ингибируют теломеразу посредством связывания с геном hTERT
Производные 1,4-имидазола	Алкилирующие и алифатические фрагменты блокируют активные участки теломеразы
Димерный имидазол	Разрушение Т-петли теломер
Иметелстат	Прямое блокирующее связывание с теломеразами
Малые интерферирующие РНК	Подавление экспрессии (сайленсинг) гена hTERT посредством РНК-интерференции
Акридин	Связывание с белками-шаперонами HSP90 с формированием лигандов — ингибиторов теломеразы
Кумарины	Подавление теломеры путем связывания с промотором C-MYC
Натуральные ингибиторы теломеразы	Оксоизоапорфин	Стабилизация теломер
Индол-3-карбинол	Подавление экспрессии мРНК hTERT
Диэтилстилбестрол	В сочетании с индол-3-карбинолом — подавление экспрессии мРНК hTERT
Натуральные активаторы теломеразы	Циклоастрагенол	Стимулирует выработку теломеразы через сигнальные пути ERK, JAK/STAT
PROX1 (гомеобоксный белок Просперо 1)	Обладает активирующей активностью к рецепторам hTERT
CDCL5 (Cell division cycle like-5)	Активация транскрипции промотора hTERT
SPT5 (Suppressor of Ty 5 homolog)	Опухолеспецифичный стимулятор теломеразы
RFPL3 (Ret Finger Protein Like 3)/CBP (cyclic AMP response element binding protein)	Совместный запуск ацетилирования RFPL3-белка и активации hTERT
Лептин	Активация hTERT посредством связывания белков STAT3 и Myc/Max/Mad между собой
Синтетические активаторы теломеразы	GRN510 (производное циклоастрагенола)	Активация экспрессии гена hTERT

Патологическая активность экспрессии TERT играет в целом наибольшую роль в развитии таких опасных опухолей, как глиобластома, меланома, рак мочевого пузыря и рак щитовидной железы [27]. Несмотря на наличие сведений о большом количестве потенциальных регуляторов теломеразы, было бы преждевременно делать выводы об их однозначной эффективности ввиду разрозненного характера процесса их изучения in vivo. Если рассматривать вышеуказанные регуляторы теломераз как потенциальные лекарственные агенты, потребуется сфокусировать внимание на их фармакологических и фармакодинамических показателях, возможных механизмах формирования резистентности и идиосинкразии. Для попытки решения данного вопроса необходима многолетняя работа по формированию обширной базы мультицентровых исследований in vitro, с последующим переходом к стадии in vitro в рамках доказательной медицины. Теломераза является далеко не единственным ключевым звеном в онкогенезе, однако изучение действия ее регуляторов на злокачественные процессы в разных типах клеток/тканей поможет добиться серьезного продвижения в понимании глубоких основ тераностики (диагностика + лечение опухолей).

## ИММУННАЯ ТЕОРИЯ

Одной из наиболее молодых теорий старения является иммунная теория, оформившаяся в начале 2000-х гг., отчасти созвучная с теломерной теорией. Утрата собственной теломеразной активности свойственна всем клеткам, проходящим специализированную дифференцировку, в т.ч. Т-лимфоцитам [28][29]. Это может приводить к более низкой устойчивости пожилых людей к различным антигенам, в частности инфекционным (вирусы, бактерии, грибы) и опухолевым (метастазы) агентам [30]. На клеточном уровне это объясняется прогрессирующим снижением интенсивности хемотаксиса, фагоцитарной активности, повышением экспрессии провоспалительных цитокинов, развитием аномалий внутриклеточного сигналинга и выработки колониестимулирующих факторов [31].

Несмотря на приоритетную роль теломераз-ассоциированных неинфекционных заболеваний (ишемическая болезнь сердца, онкологические заболевания) в танатогенезе, предпринимались попытки найти взаимосвязь между функциональным состоянием клеточного иммунитета и старением. Примером тому являютcя попытки стимуляции витальных функций лабораторных животных путем введения in vitro стволовых клеток — предшественниц гемопоэза и иммуногенеза. По итогам одной из аутотрансплантаций молодого костного мозга было неожиданно выявлено подавление выработки у подопытных животных нейросупрессоров (CCL11) и β2-микроглобулина, что способствовало повышению когнитивных функций: улучшению памяти и скорости мышления [32].

В контексте данной темы занимательным является вопрос о связи между патологией иммунного статуса и возраст-обусловленным снижением когнитивных функций. По данным исследования, проведенного в г. Сакраменто (штат Калифорния, США) среди 1337 пациентов пожилого возраста (>60 лет), отмечалась взаимосвязь между траекторией снижения когнитивных функций по данным Краткой Шкалы Оценки психического статуса (Modified Mini-Mental State Examination), с одной стороны, а также повышением базального уровня цитокинов IL-6 (β=0,0935 (95% ДИ: 0,055–0,13), TNF-α (β=0,0944 (95% ДИ: 0,032–0,157) и титром антител к цитомегаловирусу (CMV IgG) (T=0,0409 (95% ДИ: 0,013–0,069). Нельзя исключать, что подобного рода результат был достигнут за счет более эффективного распознавания и уничтожения нейротропных инфекционных агентов (в т.ч. вышеназванного цитомегаловируса, прионов). Такой неожиданный результат в перспективе может стать многообещающей основой для дальнейшего изучения и разработки цитокин-ассоциированных методов скрининга, ранней диагностики и лечения нейродегенеративных заболеваний (болезнь Альцгеймера, Паркинсона, Пика и т.д.) [33].

Несмотря на обнадеживающие результаты вышеуказанных экспериментов и важность проблемы, иммунная теория старения остается до сих недооцененной исследователями. Причиной тому является необходимость многолетних координированных усилий между представителями фундаментальной и клинической медицины разных стран. Авторы обзора из оптимистических соображений придерживаются мнения, что широкое внедрение генно-инженерных технологий станет реальностью уже в первой половине XXI в.

## ЭНДОКРИННАЯ ТЕОРИЯ

Основоположником и пионером эндокринной теории старения является французский патофизиолог Шарль Эдуар Броун-Секар (Broun-Sequard, 1817–1894), в 1889 г. определивший старение как следствие «нарушения в гормональной секреции организма на целостно-физиологическом уровне организации протоплазмы» [34]. На сегодняшний день имеются достоверные данные, что активность эндокринных желез меняется на разных этапах онтогенеза. Например, у пожилых пациентов (≥60 лет) чаще выявляется субклинический гипотиреоз с уровнем тиреотропного гормона в пределах 7–10 мЕд/л [35], снижение фракций общего и свободного тестостерона [36], а также выраженное снижение пиковой концентрации мелатонина в среднем на 50% от уровня 20-летнего возраста [37].

Другим показательным примером эндокринного старения являются менопауза (у женщин) [38][39] и андропауза (у мужчин). Наступление данного периода у женщин связано с сочетанным снижением функции тестикул и яичников, сопровождается лабораторными признаками гипергонадотропного гипогонадизма, недостаточностью секреции гормона роста и соматомединов (инсулиноподобный фактор роста (ИФР-1, -2), гиперреактивностью мозгового слоя надпочечников, повышением активности ядерных минералокортикоидных рецепторов с развитием метаболических (ожирение, сахарный диабет 2 типа), сердечно-сосудистых (дисфункция эндотелия, дислипидемия), скелетно-мышечных (остеопения, остеопороз, саркопения) и урогенитальных нарушений (генитоуринарный синдром, недержание мочи, дизурия) [38].

По мнению авторов, разработка эндокринной теории старения позволяет расширить сведения об известных на сегодняшний день гормонах, выделить ранее не изученные регуляторы жизненно важных функций. Однако проводить подобного рода исследования крайне важно параллельно с интенсивным изучением структуры и активности рецепторов у пациентов при различных эндокринных заболеваниях, так как без их существования и работы циркуляция гормонов и реализация их действия бессмысленны [39].

## ТЕОРИИ СТАРЕНИЯ КАК РЕЗУЛЬТАТ ПОВРЕЖДЕНИЙ

Повреждение ДНК — это многоуровневый процесс, затрагивающий наследственную информацию на системном (нарушения транскрипции, репликационный стресс), молекулярном (хромосомные, генные аберрации) и клеточном уровнях (сбой пролиферации стволовых клеток, повреждение митохондрий) [34].

Повреждение наследственной информации является следствием воздействия множества факторов, разных по своей природе и интенсивности. Любой дефект ДНК несет угрозу как для отдельной клетки, так и для всего организма, поэтому существуют механизмы репарации ДНК [40–46].

К непосредственным причинам повреждения ДНК in vivo и in vitro относят целый спектр биохимических превращений: повреждение активными формами кислорода [43], внутриклеточное накопление поврежденных агрегированных белков [41][42], дезамидирование [44], гликирование белков (при нарушениях углеводного обмена — более стремительно) [45], нарушение системы протеолитической системы деградации белков [46] и т.д. ДНК-повреждающие механизмы лежат также в основе ятрогенных побочных эффектов современной адъювантной терапии при онкологических заболеваниях. Многолетнее применение такого рода терапии за счет системности действия и невозможности обеспечения избирательной деструкции опухолевых клеток зачастую оборачивается повышением риска развития возраст-ассоциированных заболеваний и ухудшением прогноза для пациентов [42]. Помимо противоопухолевого эффекта аккумуляция вышеперечисленных молекулярно-генетических взаимодействий приводит к многоуровневой деградации организма: дестабилизации генома, укорочению теломер, нарушению эпигенетических механизмов, нарушениям протеостаза, повреждениям клеточных рецепторов к питательным субстратам (глюкоза, свободные жирные кислоты), митохондриальной дисфункции, нарушению межклеточной коммуникации с исходом в аноикис и т.д. [43–46]. По мнению авторов, систематизация известных и поиск новых механизмов повреждения/репарации генетического аппарата ДНК являются одними из первоочередных задач отечественной доказательной геронтологии, решение которой позволит создать должный импульс к интенсификации развития фундаментальной и клинической медицины.

## НЕКОТОРЫЕ ПОДХОДЫ К ANTI-AGE ТЕРАПИИ

Современная биологическая наука обладает широким арсеналом методов изучения процессов старения и разработки лечебно-профилактических стратегий.

Одним из перспективных направлений представляется разработка anti-age терапии мезенхимальными стволовыми клетками (МСК). МСК являются группой гетерогенных клеток, обладающих способностью к мультипотентной дифференцировке [47][48]. Данный вид клеток можно экстрагировать из различных доступных тканей человека в лабораторных условиях (костный мозг, подкожно-жировая клетчатка, зубная пульпа) с последующим репрограммированием для получения различных клеток [48]. Такого рода манипуляции могут потенциально применяться для восстановления целостности органов и тканей при повреждениях различного генеза с последующим исходом в виде увеличения продолжительности жизни.

Данный вид терапии в отдельных случаях доказал эффективность при купировании особо опасных состояний, требующих реанимационной поддержки, например, при лечении острого респираторного дистресс-синдрома (ОРДС). По данным исследования 2015 г., проведенного на базе Каролингского и Уппсальского университетов (Швеция), введение 2 пациентам (58 и 46 лет соответственно) с клинической картиной ОРДС культуры МСК в расчете 2×10⁶ клеток/кг массы тела позволило добиться видимого улучшения по данным лабораторно-инструментальных исследований и благоприятного исхода заболевания [49–51]. Несмотря на сиюминутный успех экспериментального лечения ОРДС, пациенты нуждаются в продолжении научного наблюдения с целью определения шансов развития побочных эффектов (например, злокачественных новообразований, иммунодефицитов и т.д.).

Одной из потенциальных областей применения МСК является трансплантология, успешному развитию которой препятствует одно из самых опасных и жизнеугрожающих осложнений — реакция «трансплантат против хозяина» (РТПХ) [52]. Препаратами первой линии для лечения РТПХ традиционно являются глюкокортикостероиды, однако в 50% случаев развития острой формы РТПХ данная терапия оказывается неэффективной, что приводит к увеличению риска смерти в течение ближайших 2 лет у резистентных пациентов до 80%. Хронические формы РТПХ требуют назначения глюкокортикостероидов длительного действия, что предсказуемо чревато высоким риском развития побочных эффектов (язвенная болезнь, остеопороз, прибавка массы тела, нарушения углеводного обмена и т.п.). По данным Kelly K. и соавт. (2021) [52], в качестве препаратов второй линии могут рассматриваться МСК, обладающие иммуносупрессивным и иммунорегуляторным эффектами за счет выработки цитокинов, переноса митохондрий, внеклеточного переноса РНК с помощью микровезикул и экзосом. Несмотря на многообещающие возможности, МСК на сегодняшний день не могут широко применяться для лечения РТПХ, пока не продемонстрируют эффективность и безопасность в рандомизированных мультицентровых слепых плацебо-контролируемых исследованиях. Тем не менее МСК-терапия в эндокринологии потенциально может найти свое применение при целом ряде состояний, например синдроме диабетической стопы, восстановлении функции β-клеток при сахарном диабете 1 типа, надпочечниковой недостаточности, врожденной дисфункции коры надпочечников, преждевременной недостаточности яичников и т.д.

Также в научно-медицинской литературе упоминается и другой метод персонализированной anti-age терапии — трансплантация фекальной микробиоты (ТФМ) — процедура переноса кишечной микробиоты в составе фекального материала от здорового донора к больному реципиенту (эндоскопически per os/rectum) с целью восстановления баланса микрофлоры кишечника. Данный метод является золотым стандартом лечения псевдомембранозного колита, вызываемого Clostridium difficile [53]. Представители кишечной микробиоты, включающей в себя более 1014 микроорганизмов (бактерии, бактериофаги, грибы), определенным образом влияют, по современным данным, на обмен биологически активных веществ в ЦНС (ГАМК, серотонин, дофамин, гистамин, глутамат, ацетилхолин) [54] и формируют коммуникативную ось «кишечник-мозг» [55]. Наличие подобных нейровисцеральных связей дает потенциальные возможности для осуществления трансплантации микробиоты (Acidobacteria и Bifidobacterium вместо Helicobacteraceae и Desulfovibrionaceae) лицам с нейродегенеративными заболеваниями; осуществление подобной процедуры в Нанкинском университете (КНР) на трансгенных лабораторных мышах APPswe/PS1dE9 (n=16) позволило снизить уровень нейронального накопления бета-амилоида (Аβ-40, Аβ-42) и τ-фосфорилирования (p<0,01) — ключевых патогенетических звеньев болезни Альцгеймера [55]. Следует отметить, что осуществление испытаний по трансплантации микробиоты людям с целью anti-age терапии является на сегодняшний день потенциально бесперспективным с точки зрения соблюдения этических норм, создания соответствующей инфраструктуры, организации логистики и соблюдения норм безопасности.

Другим не менее перспективным методом управления старения является разработка препаратов трансмембранного белка Klotho, активно участвующего в регуляции кардиоренальных и ренокардиальных взаимодействий, углеводного и минерально-костного обмена; его концентрация снижается в связи с возрастом in vivo, а его отсутствие у нокаутных мышей вызывает преждевременную гибель [56]. В качестве методов «Klotho-терапии» предлагаются различные гипотетические методики: деметилирование промотора Klotho ингибиторами ДНК-метилтрансферазы, ингибирование деацетилаз гистонов А, активация PPAR-γ рецепторов тиазолидиндионами (пиоглитазон, троглитазон), назначение синтетических аналогов витамина D (парикальцитол) и т.д. [56]. Именно поэтому, с точки зрения авторов, разработка и изучение моделей «управления белком Klotho» является интересным с точки зрения расширения и даже возрождения научного интереса к ранее забытым/ограниченно применяемым/запрещенным лекарственным средствам при условии успешного проведения мультицентровых рандомизированных клинических исследований.

## ЗАКЛЮЧЕНИЕ

Следует признать, что среди существующих на сегодня теорий старений нет ни одной, которую можно было бы безоговорочно противопоставить другим и считать единственно правильной. Авторы придерживаются позиции, что в основе возраст-ассоциированных заболеваний лежит синергическое взаимодействие всех вышеперечисленных факторов в различном соотношении у каждого отдельно взятого индивидуума. Например, у одной группы пациентов могут преобладать эндокринные механизмы старения (за счет отягощенного анамнеза по углеводному обмену, заболеваниям щитовидной железы), у другой — патология иммунного ответа (ВИЧ-инфекции, иммунодефициты др. генеза) и т.д. Старение является результатом нарушений способности наследственного материала клеток и тканей к репарации в результате накопления генетических, эпигенетических, теломерных, эндокринных и иммунных аномалий в процессе онтогенеза. Развитие концепции персонализированной медицины требует не только углубленного изучения фундаментальных вех, но также создания и испытания лекарственных средств в соответствии с догмами доказательной медицины. Изучение омиксных (геномных, протеомных и метаболомных) механизмов старения и методов их управления необходимо для решения насущных проблем современного отечественного и мирового здравоохранения.

## ДОПОЛНИТЕЛЬНАЯ ИНФОРМАЦИЯ

Источник финансирования. Исследование проводится в рамках Государственного задания: «Влияние эпигенетических факторов на течение менопаузы у женщин с эндокринопатиями аутоиммунного генеза в рамках формирования модели “здорового старения”», регистрационный номер АААА-121030100033-4.

Конфликт интересов. Авторы декларируют отсутствие явных и потенциальных конфликтов интересов, связанных с публикацией настоящей статьи.

Участие авторов. Михеев Р.К., Оплетаева О.Н. — концепция и дизайн исследования; Михеев Р.К., Григорян О.Р., Шереметьева Е.В., Абсатарова Ю.С. — сбор и обработка материала; Михеев Р.К., Одарченко А.С., Григорян О.Р. — написание текста; Андреева Е.Н., Григорян О.Р. — редактирование текста. Все авторы одобрили финальную версию статьи перед публикацией, выразили согласие нести ответственность за все аспекты работы, подразумевающую надлежащее изучение и решение вопросов, связанных с точностью или добросовестностью любой части работы.
